# Dual-Target Inhibition of CDK5 and PBK in Pituitary Neuroendocrine Tumors: Mechanisms and Therapeutic Potential

**DOI:** 10.3390/genes17080909

**Published:** 2026-07-31

**Authors:** Jinghao Jin, Zhaoyi Yi, Hongyun Wang, Lei Gong, Yazhuo Zhang, Weiyan Xie

**Affiliations:** Beijing Neurosurgical Institute, Capital Medical University, Beijing 100070, China; jinjinghao@mail.ccmu.edu.cn (J.J.); yizhao_yi@163.com (Z.Y.); 77why@sina.com (H.W.); pandora3118@163.com (L.G.)

**Keywords:** PitNETs, reciprocal phosphorylation, molecular biomarker, candidate dual-target inhibitor, AI-based drug screening

## Abstract

Background/Objectives: Pituitary neuroendocrine tumors (PitNETs) frequently exhibit invasive behaviors that complicate clinical treatment. While cyclin-dependent kinase 5 (CDK5) and lymphokine-activated killer T-cell-originated protein kinase (PBK, also known as PDZ-binding kinase) are implicated in tumor progression, their reciprocal regulatory mechanism remains unclear. This study aims to elucidate the CDK5-PBK interaction in PitNETs and identify potential therapeutic agents targeting this pathway. Methods: We utilized proximity labeling and phospho-specific assays to characterize the CDK5 and PBK interaction in PitNET cell lines. Immunohistochemical analysis was performed on patient tumor tissues to evaluate clinical relevance. Artificial intelligence (AI)-based virtual screening was employed to discover dual-target inhibitors. The therapeutic efficacy of the identified compound, proguanil hydrochloride, was subsequently evaluated using in vitro functional assays, alongside in vivo xenograft animal models. Results: We identified a mutual phosphorylation loop between CDK5 (at S159) and PBK (at T9) that activates insulin signaling, thereby promoting cellular proliferation and invasion in PitNETs. Patient tumor analysis revealed that the co-expression of phosphorylated CDK5 (S159) and PBK (T9) significantly correlates with tumor invasiveness (*p* < 0.001). Through AI screening, proguanil hydrochloride was identified as a candidate dual-target inhibitor. In vitro assays confirmed that it effectively reduces tumor cell growth, while in vivo xenograft studies validated its capacity to inhibit tumor progression. Conclusions: The CDK5-PBK mutual phosphorylation axis serves as a key driver of invasiveness in PitNETs. Proguanil hydrochloride represents a promising candidate dual-target therapeutic agent capable of disrupting this pathway to suppress tumor growth.

## 1. Introduction

PitNETs represent the second most common intracranial tumors, accounting for 17.4% of all primary central nervous system tumors [1]. Among these, 35–60% exhibit invasive behavior, infiltrating the dura mater, bones, or adjacent structures such as the cavernous or sphenoid sinuses. These invasive PitNETs (IPitNETs) pose significant clinical challenges due to their low rate of complete surgical resection and higher recurrence rates, severely impacting patient survival and prognosis [2,3,4]. Despite their prevalence, the molecular drivers of PitNET pathogenesis remain poorly understood.

Recent work highlights protein kinases as critical regulators of tumor behavior, modulating cellular processes through intricate phosphorylation networks. Among these, CDK5 and PBK emerged as key mediators of pituitary tumor aggressiveness in our prior studies [5,6,7]. Crucially, building on this foundation, our recent work revealed a novel reciprocal phosphorylation relationship between CDK5 and PBK—demonstrating bidirectional regulation that potently drives tumor progression.

This discovery of reciprocal CDK5-PBK phosphorylation presents a compelling therapeutic target for PitNETs. Yet translating such molecular insights into effective treatments faces significant hurdles. Pharmacotherapy for prolactinomas remains constrained: Dopamine agonists, the first-line therapy for prolactinomas, elicit suboptimal responses or resistance in 10–30% of patients [8,9,10]. For other PitNET subtypes, pharmacological options are largely restricted to adjuvant roles post surgery or salvage therapies, underscoring a critical unmet clinical need for novel strategies to overcome resistance and broaden treatment efficacy across subtypes [11].

To directly address this gap by targeting the CDK5/PBK signaling axis, we pursued the screening and identification of novel inhibitory compounds. Artificial intelligence (AI) offers transformative potential for accelerating drug discovery in this context [12,13,14]. Deep learning models (GCNs, GATs) combined with large chemical databases (ChEMBL) excel at predicting multi-target kinase interactions, enabling efficient screening [15,16,17]. Similarly, emerging AI-driven techniques and proximity labeling enhance traditional methods for mapping kinase network dynamics. Within this framework, our study integrates AI-driven discovery to identify agents disrupting the CDK5-PBK reciprocal phosphorylation loop. Collectively, these findings highlight a critical bottleneck in PitNET management: the lack of effective pharmacotherapies necessitates exploring novel therapeutic paradigms. These findings reveal a functional interdependence between CDK5 and PBK through reciprocal phosphorylation. Consequently, pharmacologic blockade targeting both kinases represents a rational therapeutic strategy with potential to overcome treatment resistance in PitNETs.

## 2. Materials and Methods

### 2.1. Cell Culture

The rat pituitary tumor cell lines GH3 (ATCC CCL-82.1^TM^; Manassas, VA, USA) and MMQ (ATCC CRL-10609^TM^; Manassas, VA, USA) were obtained from the American Type Culture Collection. These cells were cultured in Ham’s F-12K (Kaighn’s) Medium (Gibco, Waltham, MA, USA, 21127030) supplemented with 2.5% fetal bovine serum (FBS, Gibco, Waltham, MA, USA, 10100-147), 15% horse serum (HS, Gibco, Waltham, MA, USA, 16050-122), and 100 U/mL penicillin/streptomycin (Gibco, Waltham, MA, USA, 15070-063). Cultures were maintained in a humidified incubator at 37 °C with 5% (*v*/*v*) CO_2_.

### 2.2. shRNA Knockdown Cell Lines

Short hairpin RNA (shRNA) sequences targeting rat *Cdk5* (NM_080885) and *Pbk* (NM_001079937) were cloned into the hU6-MCS-CBh-gcGFP-IRES-puromycin lentiviral vector. The lentivirus was packaged and used to transduce GH3 and MMQ cells at a multiplicity of infection (MOI) of 20 in the presence of 5 μg/mL polybrene. At 72 h post infection, transduced cells were selected with puromycin (GH3: 5 µg/mL; MMQ: 0.5 μg/mL) for 14 days. Knockdown efficiency was validated by Western blotting.

### 2.3. TurboID-Based Neighboring Biotin Labeling Technology

The TurboID-based neighboring biotin labeling method was adapted from the protocol described by Branon et al. [18]. GH3 cells were engineered to express TurboID-CDK5 and TurboID-PBK fusion proteins. The experiment was designed with two groups: a control group and an experimental group. The control group consisted of TurboID fusion-expressing cells incubated without biotin (bait-only control), while the experimental group included the same cells incubated with biotin. Cells were cultured in T75 flasks using Ham’s F-12K (Kaighn’s) medium (Gibco, Waltham, MA, USA, 21127030) supplemented with 2.5% fetal bovine serum (FBS, Gibco, Waltham, MA, USA, 10100-147), 15% horse serum (HS, Gibco, Waltham, MA, USA, 16050-122), and varying concentrations of biotin (0, 50, 100, 200, and 500 µM). After 10 min of labeling, the cells were collected for sample preparation.

### 2.4. Cell Proliferation Assay

Cells were plated in 96-well plates at a density of 2 × 10^4^ cells per well. Following a 24 h culture period, CCK-8 reagent (cat. no. C6050, NCM Biotech, Suzhou, China) was introduced to the wells. The cells were then incubated at 37 °C for 2 h. Cell viability was assessed by measuring the absorbance at 450 nm using a microplate reader (BioTek Synergy H1; BioTek Instruments, Inc., Winooski, VT, USA).

### 2.5. Colony Formation Assay

In the colony formation assay, cells were plated at a density of 200 cells per well in 6-well plates and cultured for 14 days. After fixation with 4% paraformaldehyde, the colonies were stained with crystal violet and subsequently visualized. The number of colonies was then quantified using ImageJ software (version 1.54g).

### 2.6. Western Blot (WB)

Cells or tissue homogenates were lysed in RIPA buffer (cat. no. WB3100, NCM Biotech, Suzhou, China) containing ProtLytic Protease and Phosphatase Inhibitor Cocktail (cat. no. P002, NCM Biotech). The separated proteins were then transferred to a PVDF membrane (cat. no. IPVH00010, Millipore, Carrigtohill, Ireland) using a transfer system (cat. no. SVT-2, Wuhan Servicebio Technology Co., Ltd., Wuhan, China). After blocking with NcmBlot blocking buffer (cat. no. P30500, NCM Biotech), the membranes were incubated with primary antibodies at 4 °C overnight. The primary antibodies used are listed in Supplementary Table S1. The membranes were then incubated with secondary HRP-conjugated antibodies (cat. nos. SA00001-1 and SA00001-2; Proteintech Group, Inc., Rosemont, IL, USA; 1:3000) for 2 h at room temperature. The protein bands were visualized using NcmECL Ultra (cat. no. P10300, NCM Biotech) and detected with the ChemiDoc XRS+ System (Bio-Rad Laboratories, Hercules, CA, USA). The relative intensities of the bands were quantified using ImageJ software.

### 2.7. LC-MS/MS Analysis

Proteins bound to streptavidin magnetic beads were reduced with 4 mM dithiothreitol (DTT), alkylated with 10 mM iodoacetamide (IAA), and digested on beads with sequencing-grade trypsin. Peptides were desalted using C18 StageTips, vacuum-dried, and resuspended in 0.1% formic acid/3% acetonitrile. Tryptic peptides were analyzed using a quadrupole Orbitrap mass spectrometer (Orbitrap Exploris 480, Thermo Fisher Scientific, Bremen, Germany) coupled to an EASY-nLC 1200 ultra-high-pressure liquid chromatography system (Thermo Fisher Scientific) through a nano-electrospray ion source. Separation was performed on a 75 μm ID column packed with ReproSil-Pur 1.9 μm C18 resin using a 120 min linear gradient. Mass spectra were acquired in data-dependent acquisition mode (MS1 resolution 60,000, *m*/*z* 350–1800). Raw data were searched against the rat UniProt database. Peptide- and protein-level FDRs were controlled at <1%.

### 2.8. Phos-Tag SDS-PAGE

Phos-tag SDS-PAGE was conducted following the manufacturer’s instructions. In brief, during the preparation of the 12.5% separating gel for SDS-PAGE, 50 μM Phos-tag acrylamide (cat. no. AAL-107; FUJIFILM Wako Pure Chemical Corporation, Osaka, Japan) and 50 μM MnCl2 (cat. no. C0210510223; Nanjing Chemical Reagent Co., Ltd., Nanjing, China) were incorporated. Following electrophoresis, the gel was washed with transfer buffer (cat. no. G2017; Wuhan Servicebio Technology Co., Ltd., Wuhan, China) containing 10 mM EDTA (cat. no. E1170, Wuhan Servicebio Technology Co., Ltd., Wuhan, China) for 10 min, and the proteins were subsequently transferred to a PVDF membrane for Western blot analysis.

### 2.9. Dephosphorylation Assay

Briefly, the protein sample was diluted with H_2_O to a final volume of 50 μL containing 1× NEBuffer for Protein MetalloPhosphatases (PMPs) and 1 mM MnCl_2_. Lambda Protein Phosphatase (1 μL, 400 units) was added, and the reaction was incubated at 30 °C for 30 min.

### 2.10. Dataset Collection and Processing

Datasets of CDK5- and PBK-associated compounds were collected from the ChEMBL database and processed as follows: (1) Compounds with only CDK5 or PBK inhibition assay data (assay type = B) were retained. (2) Molecules with detailed assay values (IC_50_, EC_50_, K_i_, and K_d_) were kept; if a compound had multiple test values, the mean value was used. Compounds with conflicting activity annotations were excluded. (3) Duplicates were removed. (4) Compounds in the CDK5 and PBK datasets were classified into inhibitors (≤1 μM) and non-inhibitors (>1 μM). In total, 1330 molecules (combined CDK5 and PBK datasets) were obtained. The datasets were divided by scaffold-based par titioning into three sub-datasets: training set (80%), validation set (10%), and testing set (10%). The training set was used to build predictive models. The testing set was employed to optimize hyperparameters, and the validation set was used to evaluate the predictive ability of the established models. RNA sequencing was performed on an Illumina NovaSeq 6000 platform (paired-end 150 bp). Reads were aligned to the rat genome (rn6) using HISAT2, and gene-level counts were obtained with featureCounts. Differentially expressed genes were identified with DESeq2 (|log_2_ fold change| ≥ 1, *p* < 0.05). KEGG enrichment was performed using clusterProfiler and GSEA with 1000 permutations (*p* < 0.05).

### 2.11. Model Construction and Evaluation

Four classical machine learning (CML) algorithms—logistic regression (LR), random forest (RF), support vector machine (SVM), and extreme gradient boosting (XGBoost)—and three deep learning (DL) algorithms—graph convolutional network (GCN), graph attention network (GAT), and AttentiveFP—were applied to build classification models for predicting the inhibitory activity of CDK5 and PBK inhibitors. SVM, LR, and RF models were established using scikit-learn (version 0.24.1; https://github.com/scikit-learn/scikit-learn; accessed on 1 June 2026). XGBoost models were constructed using the XGBoost Python package (version 1.3.3; https://github.com/dmlc/xgboost; accessed on 1 June 2026). The DL-based models were developed using the DeepChem Python package (version 2.5.0; https://github.com/deepchem/deepchem; accessed on 1 June 2026). Grid search was used to optimize the hyperparameters for each predictive model.

### 2.12. Xenograft Experiments

All animal protocols were approved by the Ethics Committee of Beijing Tiantan Hospital Affiliated to Capital Medical University and conducted in adherence to the 3R principles. Four-week-old male BALB/c nude mice (housed under specific pathogen-free conditions with ad libitum access to food and water) were utilized, with a sample size of five mice per group. Animals were allocated to experimental groups using a random number generator. Investigators were blinded to group allocation during drug administration and tumor measurement.

For all xenograft models, each mouse received a subcutaneous injection in the right axilla comprising 1 × 10^6^ GH3 cells suspended in 100 μL of serum-free medium. To evaluate the impact of gene knockdown, twenty mice were inoculated with GH3 cells stably transfected with shMock, sh*Pbk*, sh*Cdk5*, or sh*Pbk*/*Cdk5*.

In a separate pharmacological efficacy evaluation, ten mice were inoculated with untransfected GH3 cells. One week post inoculation, this cohort was randomized into a vehicle control group and a treatment group. The treatment group received intraperitoneal injections of proguanil hydrochloride at a dose of 5 mg/kg every two days. The drug was dissolved in a vehicle consisting of 5% dimethyl sulfoxide (DMSO) and 95% saline, which was identically administered to the control group. Throughout the experiment, body weight, behavior, and tumor condition were monitored regularly. Humane endpoints included >20% body weight loss, tumor ulceration, or sustained signs of distress.

Twenty days following the initial cell injection, all mice were euthanized via cervical dislocation. The tumors were subsequently excised, photographed, and the final tumor weights were strictly quantified to evaluate the in vivo tumor burden.

### 2.13. Immunohistochemistry Techniques

Prior to immunohistochemistry (IHC), tissue microarray (TMA) slides were stained with hematoxylin and eosin to assess quality and tumor content. The digitally scanned slides were analyzed using QuPath software, version 0.5.1 [19]. For dual immunofluorescence, tissue microarray slides were subjected to sequential manual staining with anti-p-CDK5(S159) and anti-p-PBK(T9) primary antibodies, followed by species-specific fluorophore-conjugated secondary antibodies. Nuclei were counterstained with DAPI. Single-cell segmentation and fluorescence intensity quantification were performed using QuPath software (version 0.5.1). Double-positive cells were defined as those exceeding the 50th percentile (median) of the fluorescence intensity distribution of all cells identified by QuPath across the entire TMA for each individual channel.

The TMA comprised 73 clinical PitNET specimens (*n* = 73). Tumor volume was calculated from preoperative MRI. Invasiveness was assessed using the Knosp grading system.

### 2.14. Patient-Derived Primary Tumor Cell Viability Assay

For primary tumor cell viability assays, freshly resected human PitNET specimens were mechanically and enzymatically dissociated. Single-cell suspensions were plated in 96-well plates and treated with graded concentrations of proguanil hydrochloride for 48 h. Cell viability was assessed using the CCK-8 assay. Dose–response curves were fitted using a four-parameter logistic regression model to determine IC_50_ values.

### 2.15. Drug Treatment

Proguanil hydrochloride (TargetMol Chemicals Inc., Wellesley Hills, MA, USA; cat. no. T4382) was dissolved in dimethyl sulfoxide (DMSO) to prepare a 10 mM stock solution. The stock was aliquoted and stored at −20 °C. Working solutions were prepared by diluting the stock in culture medium immediately before use. The final DMSO concentration was maintained at less than 0.1% in all treatment groups, and equivalent concentrations of DMSO were used as vehicle controls. Cells were treated with proguanil hydrochloride at final concentrations of 0, 2.5, 5, and 10 μM for 24 h unless otherwise specified. For the rescue experiments, Acetylcysteine (NAC; MedChemExpress (MCE), Monmouth Junction, NJ, USA; cat. no. HY-B0215) was dissolved in phosphate-buffered saline (PBS) to prepare a 5 mM working solution, and Z-VAD-FMK (Z-VAD(OH)-FMK; MedChemExpress (MCE), Monmouth Junction, NJ, USA; cat. no. HY-16658B) was dissolved in DMSO to prepare a 50 μM working solution. Cells were pre-treated with NAC (5 mM) or Z-VAD-FMK (50 μM) for 2 h, followed by incubation with 10 μM proguanil hydrochloride for an additional 24 h.

### 2.16. ROS Detection Assay

Intracellular reactive oxygen species (ROS) levels were measured using flow cytometry. GH3 cells (1 × 10^6^ cells per condition) were treated with 10 μM proguanil hydrochloride or an equivalent volume of DMSO (vehicle control) for 24 h. Following treatment, cells were harvested, washed with PBS, and incubated with the ROS-sensitive fluorescent probe, CellROX Deep Red Reagent (Thermo Fisher Scientific, cat. no. C10422), at a final concentration of 5 μM for 30 min at 37 °C in the dark. Cells were then washed three times with PBS and immediately analyzed as live cells without fixation using a BD Accuri C6 flow cytometer (BD Biosciences, San Jose, CA, USA). The probe was excited with a 640 nm red laser, and fluorescence emission (peak ~665 nm) was collected in the FL4 channel (675/25 nm bandpass filter). Singlets were gated on FSC-A vs. FSC-H, and debris was excluded based on FSC/SSC. A total of 10,000 events were acquired per sample. Data were analyzed using FlowJo software (version 10.4; FlowJo LLC, Ashland, OR, USA).

### 2.17. Statistical Analysis

Statistical analyses were performed using GraphPad Prism 9.0 and SPSS 26.0. Sample sizes were determined through power analysis (power (1 − β) = 0.80, α = 0.05) based on preliminary data, with exact n-values reported in figure legends. Data are presented as the mean ± SD for normally distributed variables or medians with interquartile ranges for non-normal distributions. Group comparisons employed two-tailed *t*-tests (two groups) or ANOVA with Tukey’s post hoc test (multiple groups), Correlations between clinical variables were assessed using Spearman’s rank correlation. Normality was assessed using the Shapiro–Wilk test. Statistical significance was set at *p* < 0.05.

## 3. Results

### 3.1. Reciprocal Phosphorylation Between CDK5 and PBK in PitNET Cell Lines

**Figure 1:**
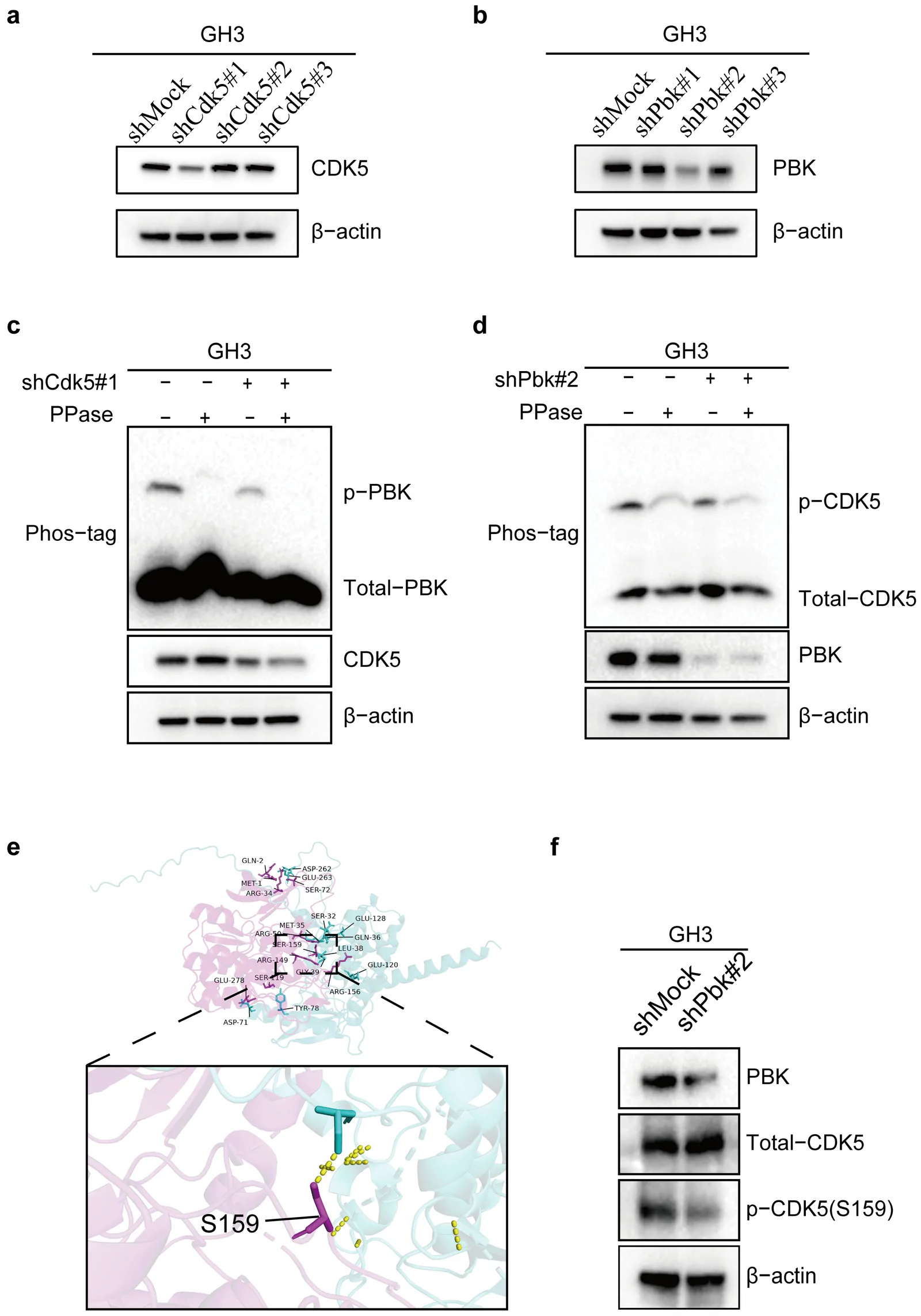
CDK5 and PBK interact via mutual phosphorylation in the GH3 pituitary tumor cell line. (**a**,**b**) The generation of stable knockdown cell lines of PBK and CDK5 in GH3 via lentivirus infection. (**c**,**d**) Reciprocal phosphorylation analysis: Phos-tag SDS-PAGE and Western blot were used to assess changes in the phosphorylation levels of the respective kinases in GH3 cells upon knockdown of CDK5 (sh*Cdk5*) or PBK (sh*Pbk*). (**e**) The AlphaFold3 complex prediction yielded an ipTM score of 0.10 and a pTM score of 0.46, indicating low confidence in the predicted protein–protein interface. Therefore, the model was used only as a hypothesis-generating tool. (**f**) Expression of p-CDK5(S159) and total CDK5 in PBK-knockdown GH3 cells.

CDK5 and PBK are both implicated in driving the progression of pituitary tumors, with prior evidence indicating that CDK5 is capable of phosphorylating PBK. Based on the screening of multiple shRNA sequences, we identified and selected the most efficient constructs for subsequent experiments: sh*Cdk5#1* and sh*Pbk#2* for GH3 cells. These specific constructs were utilized for all downstream functional and mechanistic analyses to ensure robust gene silencing (Figure 1a,b). Phosphorylation status and total protein abundance of CDK5 and PBK were assessed via Phos-tag SDS-PAGE and conventional Western blotting, respectively (Figure 1c,d).

In GH3 cells, CDK5 knockdown resulted in a marked reduction in PBK phosphorylation levels. Conversely, the silencing of PBK led to decreased phosphorylation of CDK5. These reciprocal effects suggest a potential bidirectional kinase interaction, wherein CDK5 and PBK may serve as mutual phosphorylation partners.

Our previous findings demonstrated that CDK5 phosphorylates PBK at threonine 9 (T9), thereby facilitating pituitary tumor progression [5]. Building on this, we hypothesized that PBK may in turn phosphorylate CDK5. To identify candidate phosphorylation sites on CDK5, we systematically analyzed several curated repositories, including PhosphoSitePlus (https://www.phosphosite.org; accessed on 1 June 2026), the National Center for Biotechnology Information (NCBI) Gene database (https://www.ncbi.nlm.nih.gov/gene; accessed on 1 June 2026), and the UniProt Knowledgebase (https://www.uniprot.org; accessed on 1 June 2026).

To further refine our prediction, we employed AlphaFold3-based protein structure modeling to map potential interaction interfaces between CDK5 and PBK, which yielded an ipTM score of 0.10 and a pTM score of 0.46, indicating low confidence in the predicted protein–protein interface [20]. This integrative approach identified serine 159 (S159) of CDK5 as a putative phosphorylation site targeted by PBK (Figure 1e). To experimentally validate this prediction, we examined p-CDK5 (S159) protein levels in GH3 cells following PBK knockdown. Consistent with our hypothesis, PBK silencing significantly reduced phosphorylation at the S159 site (Figure 1f).

Taken together, these findings, combined with previous evidence of a robust interaction between CDK5 and PBK, support a model in which S159 of CDK5 is regulated by PBK in a phosphorylation-dependent manner, suggesting a previously uncharacterized axis of reciprocal kinase regulation in pituitary tumorigenesis.

### 3.2. Exploring the Reciprocal Kinase Interactions and Substrate Activation Mechanisms of CDK5 and PBK Using TurboID Proximity Labeling

**Figure 2:**
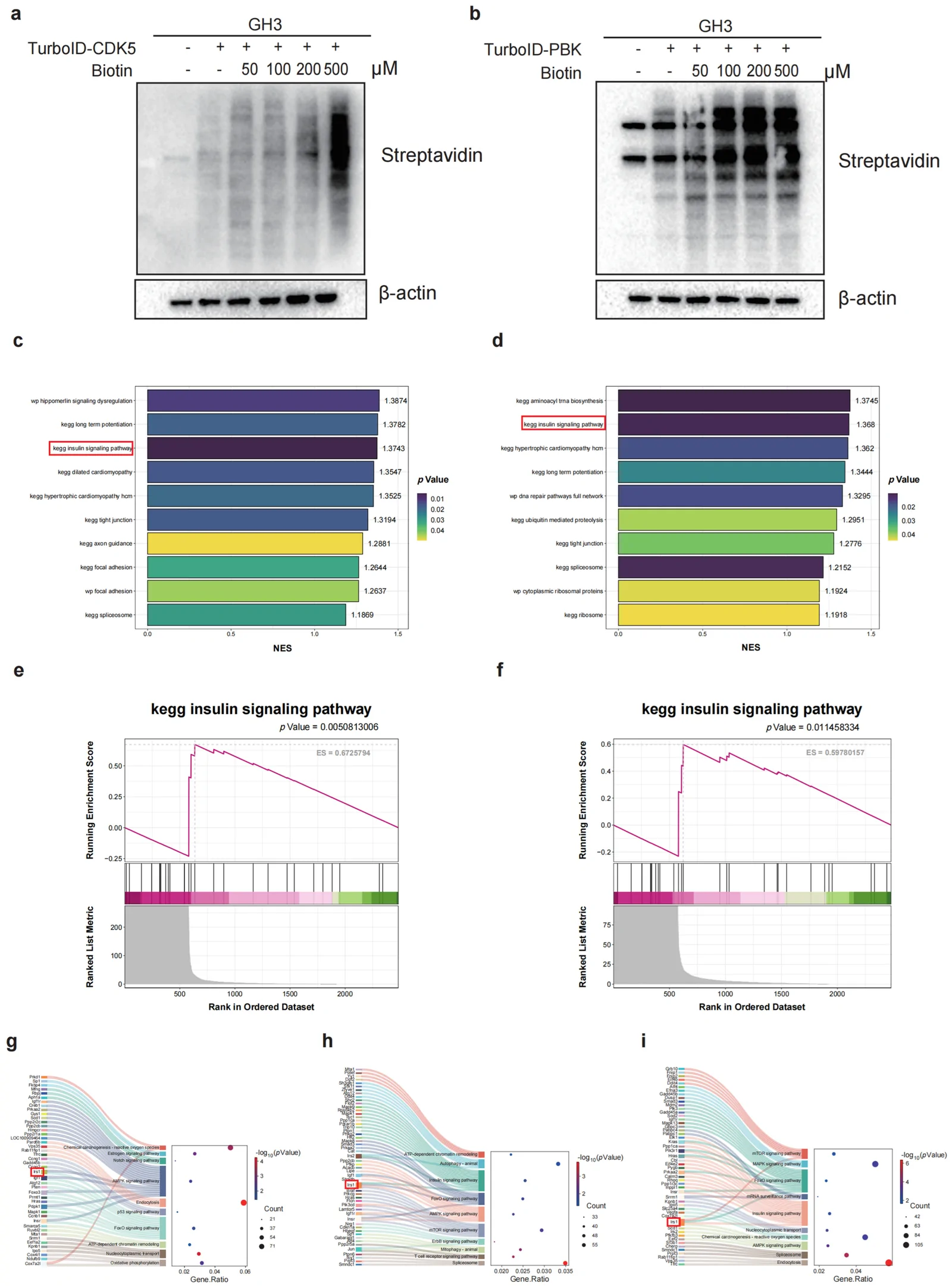
Identification of common downstream substrates of CDK5 and PBK via TurboID proximity labeling. (**a**,**b**) Validation of biotin labeling efficiency of TurboID-CDK5 and TurboID-PBK in GH3 cells by Western blot. (**c**,**d**) GSEA enrichment analysis of TurboID-CDK5 and TurboID-PBK proximal proteins in GH3 cells by mass spectrometry. (**e**,**f**) GSEA enrichment score line plots for the insulin signaling pathway. (**g**–**i**) Bubble Sankey diagrams illustrating the Kyoto KEGG pathway enrichment analysis of differentially expressed genes (DEGs) identified via RNA sequencing. The corresponding comparisons are (**g**) CDK5 overexpression versus CDK5 knockdown; (**h**) PBK overexpression versus PBK knockdown; and (**i**) concurrent overexpression of CDK5 and PBK versus concurrent knockdown of CDK5 and PBK. The red box in panels (**g**–**i**) indicates the *Irs1* gene.

To better explore the kinase substrate activation mechanisms of CDK5 and PBK, we employed TurboID proximity labeling to identify interaction proteins of these two kinases. TurboID is a highly active, promiscuous biotin ligase that can label proteins in intact live cells, with a labeling radius of approximately 15 Å from the “bait” protein fused to TurboID. We first established FLAG-TurboID-CDK5 and FLAG-TurboID-PBK overexpression models in the GH3 cell line. To determine the optimal biotin activation concentrations for proximal labeling, a gradient of 50 to 500 μM biotin was evaluated. Streptavidin-horseradish peroxidase (StrepHRP) Western blotting of crude protein extracts and streptavidin-enriched fractions demonstrated that 500 μM and 100 μM biotin optimally activated TurboID-CDK5 and TurboID-PBK, respectively (Figure 2a,b). Subsequently, proximally labeled proteins generated under these optimal conditions were isolated and identified using liquid chromatography–tandem mass spectrometry (LC-MS/MS). Gene Set Enrichment Analysis (GSEA) was performed by comparing the 100 μM biotin-activated PBK group and the 500 μM biotin-activated CDK5 group against their respective controls. The analysis revealed significant enrichment of the insulin signaling pathway, indicating that key components of this pathway potentially serve as common downstream targets for both PBK and CDK5 in GH3 cells (Figure 2c–f).

Thus, we demonstrate that CDK5 and PBK phosphorylate each other in GH3 cell lines, with IRS1 as a potential common downstream substrate (identified via proximity labeling). Using TurboID proximity labeling and LC-MS/MS, we identified IRS1 as a common proximal protein associated with both CDK5 and PBK; IRS1 is a key mediator of the insulin signaling pathway. These results underscore the bidirectional kinase interaction between CDK5 and PBK, highlighting their potential as key regulators in tumor progression.

### 3.3. Regulation of PitNET Cell Proliferation and Survival by CDK5 and PBK via the Insulin Signaling Pathway

**Figure 3:**
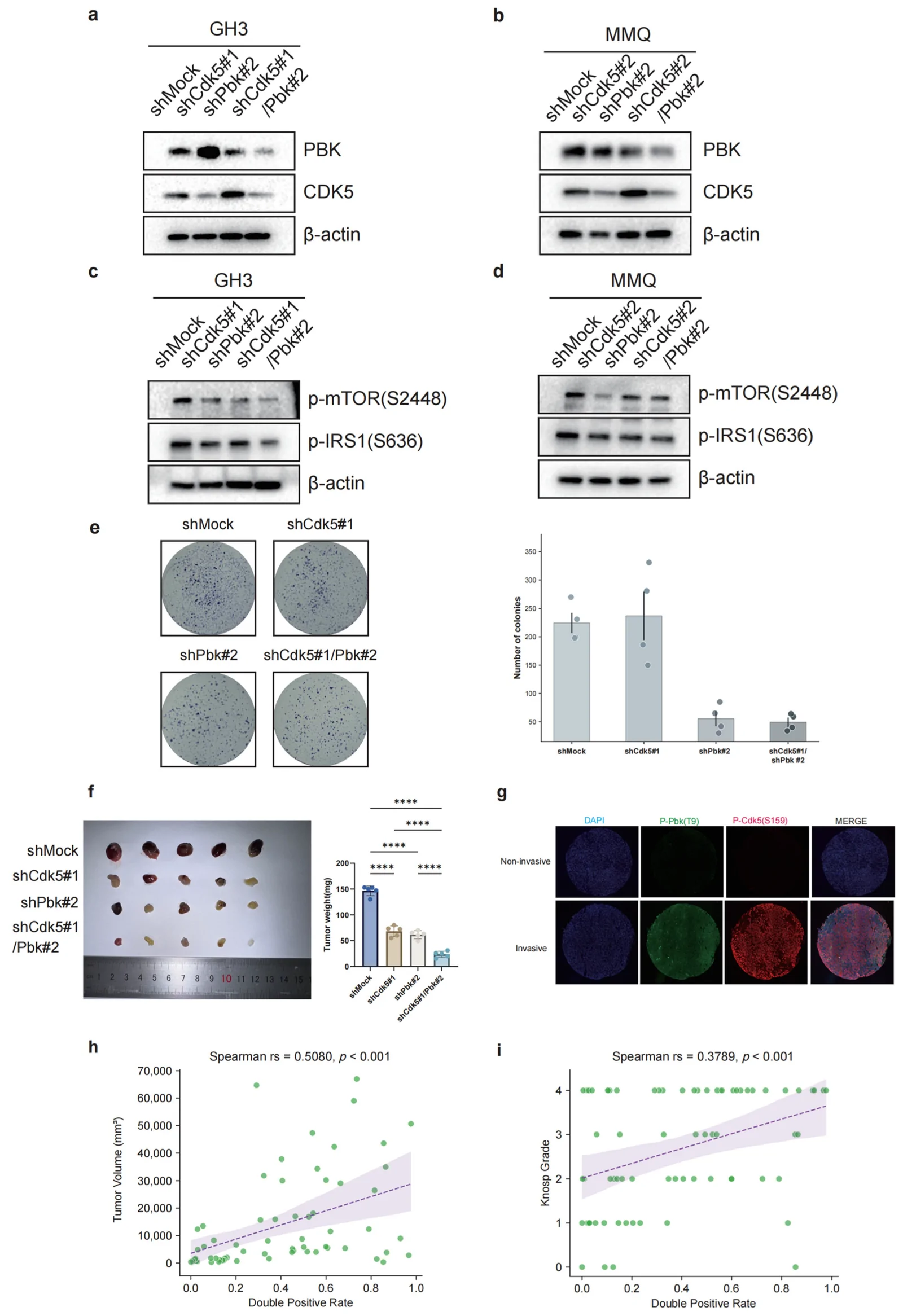
Downstream signaling pathways of CDK5 and PBK. (**a**,**b**) The generation of stable knockdown cell lines of CDK5 and PBK in GH3 and MMQ cells. (**c**,**d**) Western blot analysis of the phosphorylation of IRS1 and mTOR in indicated cells. (**e**) Colony formation assays for GH3 cells stably expressing shMock, sh*Cdk5#1*, sh*Pbk#2*, or sh*Cdk5#1*/sh*Pbk#2*. Cells were seeded in 6-well plates and cultured for 14 days, after which colonies were stained and four non-overlapping quadrants per well were imaged and counted. No inferential statistical comparison was performed for these data. (**f**) Representative images of subcutaneous tumor formation in nude mice inoculated with GH3 cells stably expressing shMock, sh*Cdk5#1*, sh*Pbk#2*, or sh*Cdk5#1*/sh*Pbk#2*. Statistical analysis of xenograft tumor weights from the shMock, sh*Cdk5#1*, sh*Pbk#2*, and sh*Cdk5#1*/sh*Pbk#2* groups (*n* = 5 per group). Both single-gene knockdowns significantly reduced tumor weight compared to the shMock control (68.14 mg and 61.62 mg vs. 146.6 mg, respectively; ****, *p* < 0.0001). The combined knockdown of CDK5 and PBK (sh*Cdk5#1*/sh*Pbk#2*) led to a further significant reduction in tumor weight compared to either the sh*Cdk5#1* (23.52 mg vs. 68.14 mg; ****, *p* < 0.0001) or sh*Pbk#2* group (23.52 mg vs. 61.62 mg; ****, *p* < 0.0001). Data are expressed as the mean ± SD. Statistical significance was determined by one-way ANOVA followed by Tukey’s multiple comparisons test. ****, *p* < 0.0001; ns, not significant. (**g**) Immunofluorescence staining of non-invasive and invasive clinical tissue samples showing DAPI (blue), phosphorylated PBK (T9, green), and phosphorylated CDK5 (S159, red). (**h**) Correlation analysis between the double-positive rate (%) of p-PBK(T9) and p-CDK5(S159) and tumor volume. Spearman’s correlation coefficient (Rs) = 0.5080, *p* < 0.001. (**i**) Correlation analysis between the double-positive rate (%) of p-PBK(T9) and p-CDK5(S159) and the Knosp tumor grade. Spearman’s correlation coefficient (Rs) = 0.3789, *p* < 0.001.

The insulin signaling pathway and its downstream effectors are essential for regulating cell growth, survival, and metabolism across various tissues. Dysregulation of these pathways, particularly through aberrant kinase activity, can drive tumor development and progression. To further elucidate the downstream signaling pathways of CDK5 and PBK, we constructed GH3 cell lines with shRNA-mediated knockdown and plasmid-based overexpression of CDK5 and PBK. The cell lines included CDK5 knockdown, PBK knockdown, combined knockdown of both CDK5 and PBK, CDK5 overexpression, PBK overexpression, and combined overexpression of both CDK5 and PBK. RNA sequencing was performed with three biological replicates for seven groups, including the control group. KEGG enrichment analysis identified the insulin signaling pathway as a principal target modulated by CDK5 and PBK (Figure 2g–i). The immunoblotting results demonstrated that CDK5 and PBK knockdown in GH3 and MMQ cell lines led to a reduction in the phosphorylation levels of key proteins, including IRS1 and mTOR (Figure 3c,d). To further investigate the functional impact of these molecular changes, we performed colony formation experiments and in vivo xenograft studies using the GH3 cell line (Figure 3e,f). Colony numbers were counted for shMock, sh*Cdk5#1*, sh*Pbk#2*, and sh*Cdk5#1*/sh*Pbk#2* groups. A reduction in colony number was observed upon CDK5 and PBK knockdown (Figure 3e). The results showed that PBK knockdown and combined CDK5/PBK knockdown reduced colony formation, whereas CDK5 knockdown alone did not show an evident reduction in the displayed data.

Taken together, CDK5 and PBK regulate key signaling pathways, particularly the insulin signaling pathway, to modulate PitNET cell proliferation and survival. Their reciprocal phosphorylation interaction affects downstream effectors such as IRS1 and mTOR, thereby contributing to tumor progression.

### 3.4. Association Between Co-Expression of p-CDK5(S159) and p-PBK(T9) with Tumor Invasiveness in Clinical Samples

We also performed dual staining for p-CDK5(S159) and p-PBK(T9) on previously collected clinical formalin-fixed paraffin-embedded tissue microarray samples (*n* = 73), followed by cell segmentation and H-score calculation using QuPath software (Figure 3g). To avoid edge effects interfering with the results, we contracted the contour of each sample by 90 µm inward to extract the regions of interest (ROIs) for further analysis. Spearman’s correlation analysis revealed significant positive associations between the p-CDK5(S159)/p-PBK(T9) dual-positive rate and tumor volume (Rs = 0.5080, *p* < 0.001; Figure 3h), as well as Knosp grade (Rs = 0.3789, *p* < 0.001; Figure 3i).

### 3.5. Identification of Dual-Target Inhibitors for CDK5 and PBK Using AI Screening and Machine Learning Models

**Figure 4:**
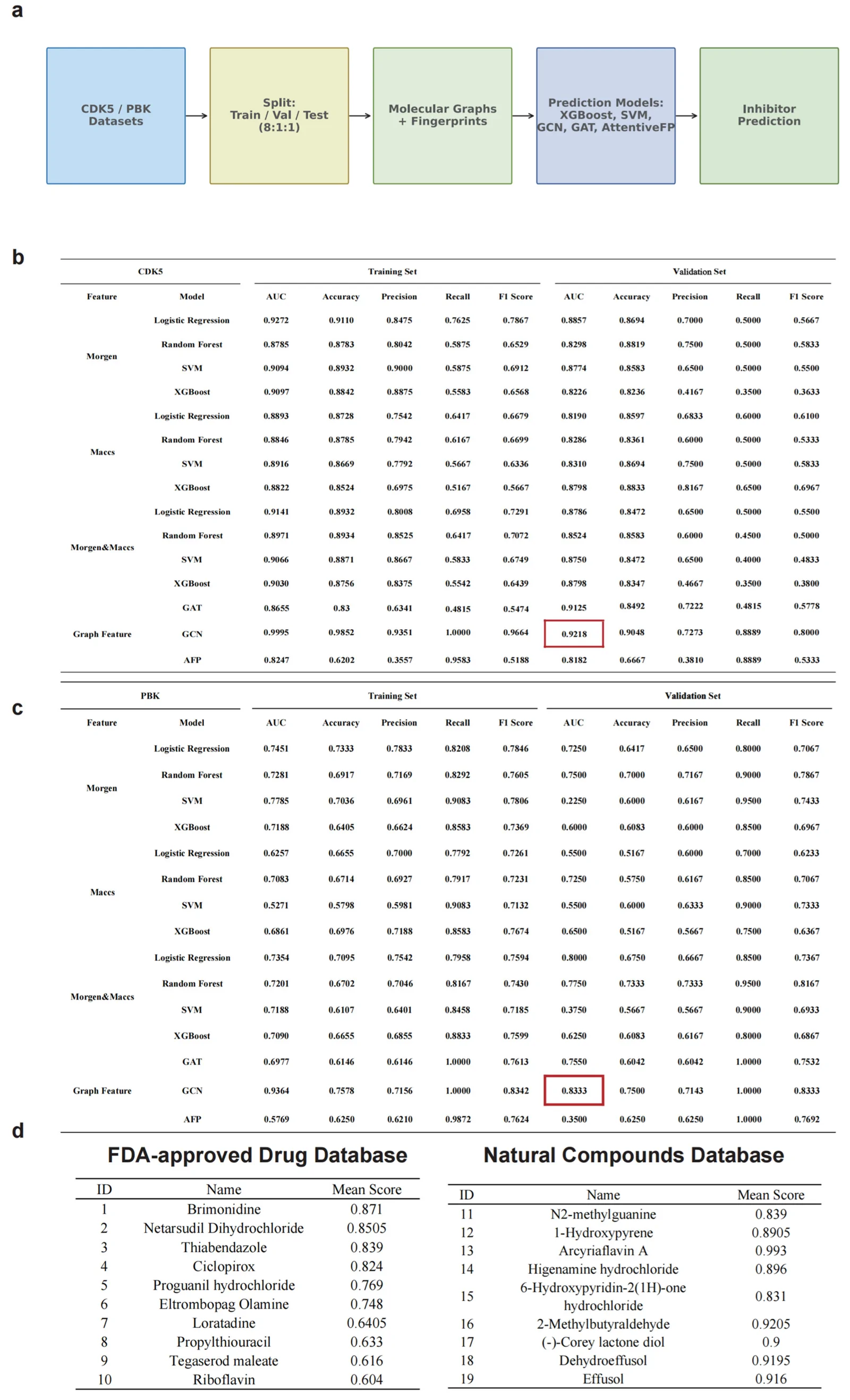
The establishment and application of a drug screening model for CDK5 and PBK inhibitors. (**a**) A schematic representation of the model construction process. (**b**,**c**) Evaluation metrics for the predictive models of CDK5 and PBK inhibitors, including AUC (area under the curve), accuracy, precision, and recall, assessed on both the training and validation datasets. The red box points to the top-performing model (highest AUC) in the validation set. (**d**) Application of the trained Graph Convolutional Network (GCN) model to infer potential dual inhibitors of CDK5 and PBK from databases of FDA-approved drugs and natural compound small molecules.

To evaluate the therapeutic potential of concurrently targeting CDK5 and PBK in the suppression of pituitary tumor proliferation, we employed an artificial intelligence-driven virtual screening strategy to identify dual inhibitors. The screening pipeline was based on curated inhibitor datasets retrieved from the ChEMBL database (https://www.ebi.ac.uk/chembl/; accessed on 1 June 2026) (Figure 4a). Our objective was to construct robust classification models capable of accurately predicting CDK5/PBK inhibitory activity and to leverage these models for the discovery of novel dual-target compounds.

A total of 15 predictive models were developed using two molecular representation paradigms—molecular fingerprints and molecular graphs—coupled with four conventional machine learning (CML) algorithms (Logistic Regression [LR], Random Forest [RF], Support Vector Machine [SVM], and Extreme Gradient Boosting [XGBoost]) and three state-of-the-art deep learning (DL) frameworks (Graph Convolutional Network [GCN], Graph Attention Network [GAT], and Attentive FP). Model-specific hyperparameters were optimized using validation set performance, and the optimal configurations are detailed in Figure 4b,c.

Model evaluation metrics—including area under the receiver operating characteristic curve (AUC), accuracy (ACC), precision, recall, and F1 score—were computed on the validation sets to quantify predictive performance and generalizability (Figure 4b,c). Specifically, the CDK5-GCN model achieved an AUC of 0.9218 on the CDK5 validation set, while the PBK-GCN model attained an AUC of 0.8333 on the PBK validation set. Subsequently, both the GCN-CDK5 and GCN-PBK models were applied to molecules from both the FDA-approved drug database and the natural-compound database. Molecules with prediction confidence scores greater than 0.6 for both CDK5 and PBK were selected. The average of the two confidence scores was calculated, and compounds were ranked accordingly; the top 10 FDA-approved drugs and the top 9 highest-scoring natural compounds were selected for further evaluation (Figure 4d).

### 3.6. Mechanism and Efficacy of OncoCompound **5** (Proguanil Hydrochloride) in Inhibiting Pituitary Tumor Cell Proliferation

Building upon our prior findings that the suppression of CDK5 and PBK attenuates pituitary tumor cell proliferation, we conducted a preliminary cytotoxicity screen of 19 OncoCompounds in MMQ and GH3 cell lines using the CCK-8 assay. Four compounds—OncoCompound **2**, OncoCompound **5**, OncoCompound **9**, and OncoCompound **13**—exhibited marked inhibitory activity in MMQ or GH3 cell lines (Supplementary Figure S1).

**Figure 5:**
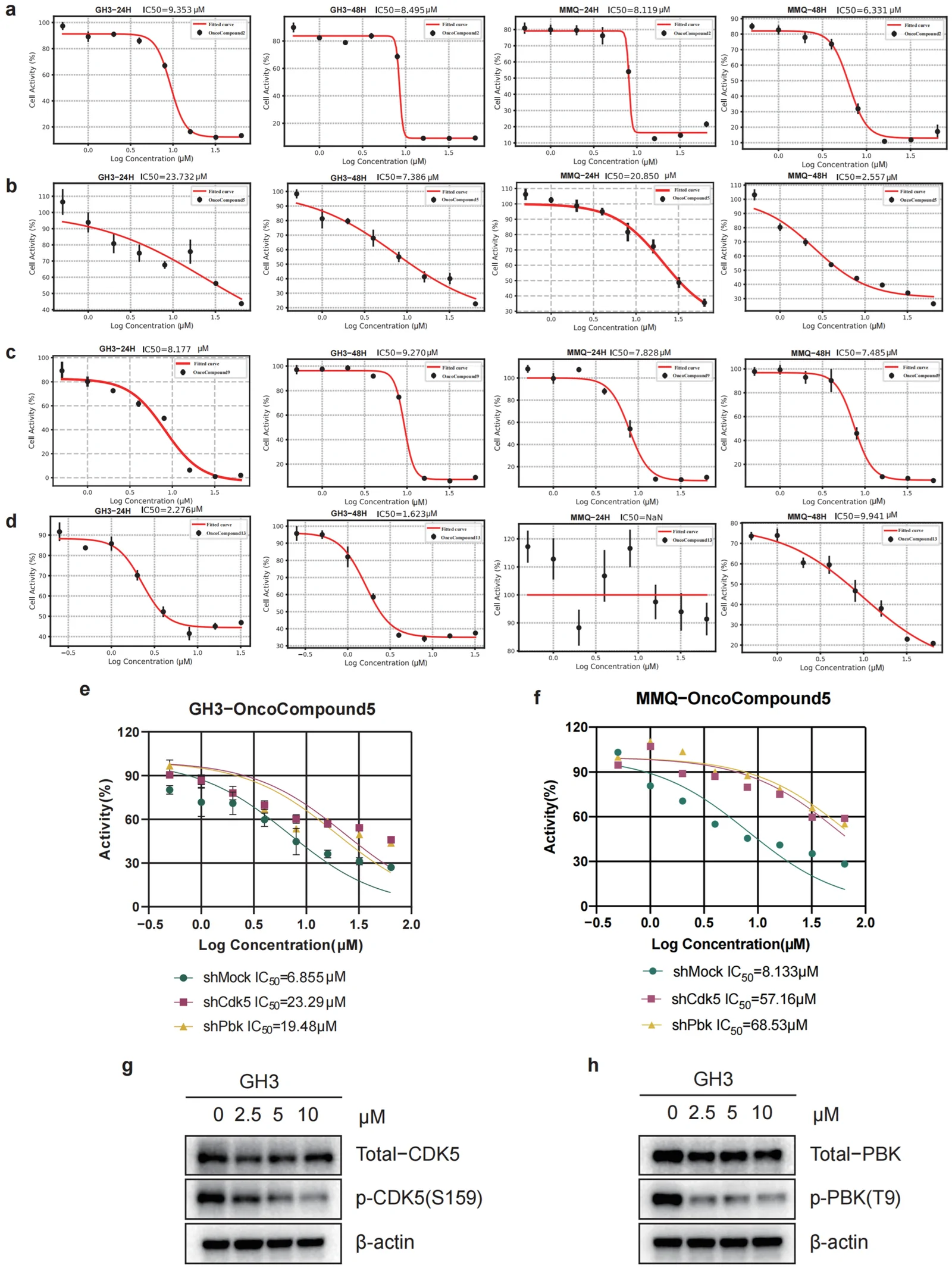
Dual CDK5–PBK targeting reduces PitNET cell viability. (**a**–**d**) Dose–response viability curves for GH3 and MMQ cells at 24 h and 48 h after treatment with four candidate inhibitors (OncoCompound **2**/**5**/**9**/**13**) show time- and dose-dependent loss of cell activity, with the mean ± SD plotted and nonlinear fits used to derive IC_50_ values;IC_50_ was not estimable for OncoCompound **13** in MMQ cells at 24 h within the tested concentration range. (**e**,**f**) shRNA knockdown of CDK5 or PBK shifts the proguanil hydrochloride curves to higher concentrations in both lines, consistent with on-target engagement. (**g**,**h**) Western blot analysis of CDK5 phosphorylation (S159) and PBK phosphorylation (T9), along with their respective total protein levels, in GH3 cells following treatment with proguanil hydrochloride at 0, 2.5, 5, and 10 μM.

Subsequently, we determined the half-maximal inhibitory concentrations (IC_50_) of these four compounds (Figure 5a–d). To further validate target specificity, IC_50_ values were reassessed in stable GH3 and MMQ cell lines with shRNA-mediated knockdown of CDK5 or PBK. Consistent with on-target engagement, depletion of either CDK5 or PBK rendered GH3 and MMQ cells less sensitive to proguanil hydrochloride, shifting the IC_50_ to higher concentrations (e.g., GH3: shMock 6.855 μM → sh*Cdk5* 23.29 μM/sh*Pbk* 19.48 μM; MMQ: shMock 8.133 μM → sh*Cdk5* 57.16 μM/sh*Pbk* 68.53 μM; Figure 5e,f). To evaluate the effects of proguanil hydrochloride on CDK5 and PBK activation, GH3 cells were treated with indicated concentrations (0, 2.5, 5, and 10 μM). Western blot analysis revealed the alteration of p-CDK5 (S159) and p-PBK (T9) levels relative to their total proteins (Figure 5g,h). Together, these complementary findings support CDK5 and PBK as pharmacological targets of proguanil hydrochloride.

**Figure 6:**
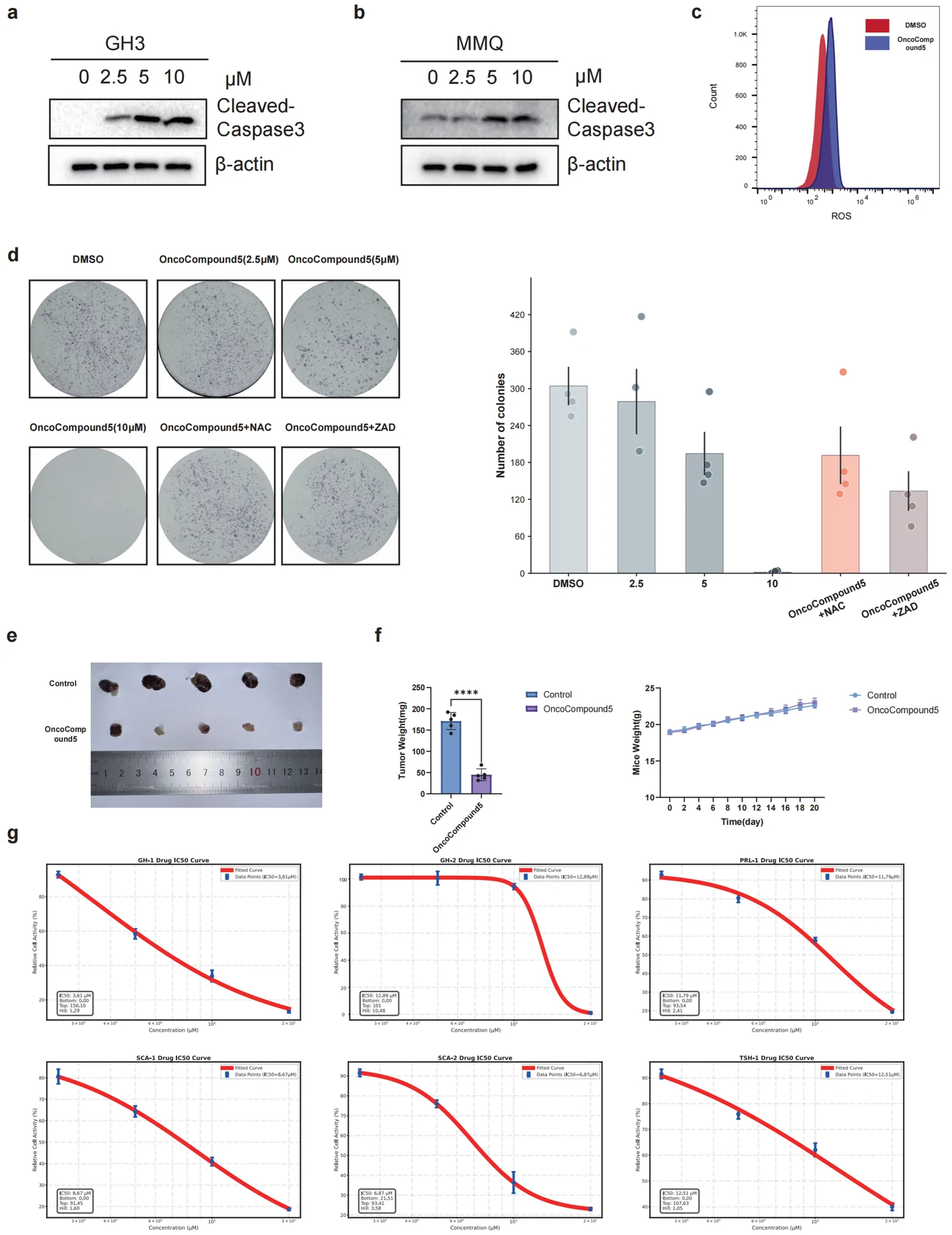
Proguanil hydrochloride induces apoptosis and ROS generation to suppress pituitary tumor growth in vitro and in vivo, exhibiting differential sensitivity across PitNET subtypes. (**a**,**b**) Western blot analysis of apoptosis markers cleaved caspase-3 in GH3 and MMQ cells treated with 0, 2.5, 5, and 10 μM of proguanil hydrochloride. (**c**) Flow cytometry analysis of reactive oxygen species (ROS) levels in GH3 cells treated with proguanil hydrochloride (blue) compared to the control group (red). (**d**) The colony formation assay showing the dose-dependent effect of proguanil hydrochloride on clonogenicity in GH3 cells. Cells were seeded in 6-well plates and treated with DMSO (control) or proguanil hydrochloride at the indicated concentrations (2.5, 5, or 10 µM) for 24 h. For the rescue experiments, cells were pre-treated with NAC (5 mM) or ZAD (50 µM) for 2 h prior to 10 µM proguanil hydrochloride exposure. After 14 days of culture, colonies were stained, and four non-overlapping quadrants per well were imaged and counted. (**e**) A representative image of GH3 xenograft tumors from control and proguanil hydrochloride-treated groups. (**f**) Tumor weight analysis showing that proguanil hydrochloride significantly reduced tumor weight compared to the control group (44.9 ± 14.0 mg versus 171.2 ± 20.2 mg, ****, *p* < 0.0001). The body weight of mice during the treatment period, indicating no significant weight loss in the proguanil hydrochloride treatment group. (**g**) IC_50_ dose–response curves for various PitNET primary cultures, including GH-1 (3.61 µM), GH-2 (12.89 µM), PRL-1 (11.79 µM), SCA-1 (8.67 µM), SCA-2 (6.87 µM), and TSH-1 (12.51 µM) tumors, highlighting differential sensitivity to proguanil hydrochloride. These findings suggest that proguanil hydrochloride effectively inhibits pituitary tumor growth in vivo and demonstrates potential for personalized therapeutic approaches.

Proguanil hydrochloride elevated cleaved caspase-3 expression (Figure 6a,b) and intracellular reactive oxygen species (ROS) levels (Figure 6c). Colony formation assays demonstrated that the dose-dependent suppression of clonogenicity induced by proguanil hydrochloride was attenuated by co-treatment with the ROS scavenger NAC or the pan-caspase inhibitor ZAD (Figure 6d). These results indicate that proguanil hydrochloride suppresses clonogenicity through ROS-dependent apoptosis, and that both oxidative stress and caspase activation contribute to this effect.

### 3.7. Proguanil Hydrochloride Suppressed Pituitary Tumor Growth In Vivo

To further assess the therapeutic potential of proguanil hydrochloride identified in vitro, we performed in vivo validation using a GH3 xenograft tumor model (Figure 6e). At twenty days post inoculation, mice treated with proguanil hydrochloride exhibited significantly reduced tumor burden compared to controls, with mean tumor weights of 44.9 ± 14.0 mg versus 171.2 ± 20.2 mg, respectively (*p* < 0.0001; Figure 6f). These results provide compelling evidence that proguanil hydrochloride exerts potent antitumor activity against pituitary neoplasms in vivo.

In parallel, we examined the cytotoxic effects of proguanil hydrochloride on freshly resected primary human PitNET specimens obtained from six patients, as summarized in Supplementary Figure S2. The cohort encompassed a spectrum of clinically and biologically distinct tumor subtypes, including two growth hormone (GH)-secreting PitNETs, one drug-resistant prolactin (PRL)-producing tumor, two silent corticotroph PitNETs (SCAs), and one thyrotropin-secreting (TSH) PitNET. Dose–response profiling revealed heterogeneity in drug sensitivity among these samples. As shown in Figure 6g, IC_50_ values ranged from 3.61 μM in GH-1 to 12.89 μM in GH-2; the PRL-1 tumor exhibited an IC_50_ of 11.79 μM, while SCA-1 and SCA-2 showed values of 8.67 μM and 6.87 μM, respectively. The TSH-1 PitNET displayed an IC_50_ of 12.51 μM.

Collectively, these findings demonstrate that proguanil hydrochloride not only suppresses pituitary tumor growth in vivo but also elicits variable cytotoxic responses across different PitNET subtypes, underscoring its potential utility in precision-targeted therapeutic strategies for pituitary tumors.

## 4. Discussion

Our study identifies a novel reciprocal phosphorylation mechanism between CDK5 and PBK that drives PitNET progression via activation of the insulin signaling/IRS1-mTOR axis. Through AI-driven drug screening, we discovered proguanil hydrochloride as a candidate dual-target inhibitor demonstrating potent antitumor activity against both CDK5 and PBK in vitro and in vivo. Importantly, we established the co-expression of phosphorylated CDK5(S159) and PBK(T9) as a clinically relevant biomarker for tumor invasiveness, effectively bridging molecular mechanisms with diagnostic applications.

This work provides evidence of bidirectional kinase cross-regulation in PitNETs, achieved through an innovative integration of proximity labeling, multi-omics profiling, and deep learning-based drug discovery. The identification of a candidate dual-target inhibitor may address the critical limitation of compensatory kinase activation observed in conventional single-target therapies.

Despite advancements in surgical management, a subset of PitNETs—particularly invasive prolactinomas—remain refractory to dopamine agonists and lack effective targeted therapies [8,11]. Although CDK5 and PBK have been individually implicated in tumorigenesis, their interaction network and downstream effectors were poorly defined. Our systematic mapping of the CDK5-PBK-IRS1 signaling hub and its role in metabolic reprogramming fills this knowledge gap. However, the spatiotemporal regulation of CDK5(S159)/PBK(T9) phosphorylation during invasion and the potential crosstalk with hormonal pathways require further elucidation.

While classified as benign tumors, aggressive PitNETs frequently exhibit high proliferation indices and invasive growth patterns that impede complete resection, leading to recurrence and poor clinical outcomes [11,21,22]. Tumor heterogeneity underscores the necessity to delineate signaling pathways governing invasiveness. CDK5 mediates tumor progression through phosphorylation of diverse substrates including FAK, GIV, PBK, and PPARγ, while also regulating glucocorticoid receptor transcriptional activity—a finding with therapeutic implications [7,23,24,25]. Our prior work demonstrated CDK5-mediated PBK(T9) phosphorylation correlating with prolactinoma invasiveness [5]. The therapeutic potential of PBK inhibition is further supported by its overexpression in malignancies such as lung adenocarcinoma (often co-occurring with CEP55 and EZH2), establishing PBK as a promising pan-cancer target [6,26]. Building on these foundations, we confirmed reciprocal CDK5-PBK phosphorylation and identified their functional convergence on insulin signaling through TurboID-mediated interactome mapping, revealing their cooperative role in PitNET aggressiveness. The significant correlation between p-CDK5(S159)/p-PBK(T9) co-expression and both tumor volume (Rs = 0.5080) and Knosp grading (Rs = 0.3789) positions this biomarker combination as a clinically actionable predictor of invasiveness. These mechanistic insights establish a rationale for dual-kinase targeting strategies.

AI-driven drug discovery has revolutionized oncology by enabling rapid evaluation of chemical libraries and molecular interactions [12,13,27,28,29]. While traditional CDK5 inhibitors suffer from off-target effects due to broad CDK family activity, and PBK inhibitors show variable efficacy across malignancies, our AI screening of FDA-approved drugs and natural compounds identified proguanil hydrochloride as a novel dual-kinase inhibitor. This repurposed antimalarial agent demonstrates potent CDK5/PBK inhibition and previously reported anti-breast cancer activity, providing potential translational relevance for aggressive PitNETs.

A limitation of this study is the need for further investigation into the specific role of CDK5 at the S159 site and PBK at the T9 site in promoting the insulin signaling pathway. Future studies should aim to elucidate the precise molecular mechanisms underlying their contribution to this pathway. Additionally, incorporating more inhibitory experiments using primary cells could provide stronger evidence for the clinical value of the identified drug candidates. These efforts would enhance the translational relevance of our findings and warrant further investigation across molecular and clinical PitNET subtypes.

## 5. Conclusions

This study reveals a reciprocal phosphorylation relationship between CDK5 and PBK, which critically regulates downstream signaling pathways, including IRS1 and the mTOR axis, in PitNETs. Co-expression of p-CDK5(S159) and p-PBK(T9) was significantly associated with increased tumor invasiveness, suggesting their potential as biomarkers for aggressive PitNET phenotypes. Furthermore, AI-based screening identified proguanil hydrochloride as a candidate dual-target inhibitor of CDK5 and PBK, demonstrating significant antitumor effects by suppressing the insulin signaling/IRS1-mTOR axis. These findings provide novel insights into the molecular mechanisms driving PitNET progression and propose a promising therapeutic strategy targeting the CDK5-PBK axis.

## Data Availability

The datasets generated and/or analyzed during the current study are available from the corresponding authors upon reasonable request. All code used in this study is publicly available at https://github.com/Timksss2/code (accessed on 1 June 2026).

## Supplementary Materials

The following supporting information can be downloaded at: https://www.mdpi.com/article/10.3390/genes17080909/s1, Figure S1: The cell viability of GH3 and MMQ cells after 24 h treatment with a panel of OncoCompounds across three concentrations; Figure S2: Comprehensive characterization of patient-derived primary pituitary tumor cultures, including TSH-1, SCA-2, PRL-1, GH-1, GH-2, and SCA-1. Supplementary Table S1: A list of antibodies used in this study; quality assessment metrics for the AlphaFold-predicted protein complex models, including the predicted template modeling score (pTM) and interface predicted template modeling score (ipTM).
